# Antibacterial suture vs silk for the surgical removal of impacted lower third molars. A randomized clinical study

**DOI:** 10.4317/medoral.20721

**Published:** 2015-11-30

**Authors:** Sergi Sala-Pérez, Marta López-Ramírez, Milva Quinteros-Borgarello, Eduardo Valmaseda-Castellón, Cosme Gay-Escoda

**Affiliations:** 1 DDS. Resident of the Master of Oral Surgery and Implantology. School of Dentistry, University of Barcelona, Spain; 2 DDS. Associate Professor of Oral Surgery. Professor of the Master of Oral Surgery and Implantology. School of Dentistry, University of Barcelona, Spain. Researcher of the IDIBELL Institute; 3 DDS, PhD. Professor of Oral Surgery, Professor of the Master in Oral Surgery and Implantology, School of Dentistry, University of Barcelona, Spain. Researcher of the IDIBELL Institute; 4 DDS, MD, PhD. Chairman and Profesor, Department of Oral and Maxillofacial Surgery, Director of the Master of Oral Surgery and Implantology, School of Dentistry, University of Barcelona, Spain. Head of the Department of Oral Surgery, Implantology and Maxillofacial Surgery, and Co-director of the Temporomandibular Joint Disease and Orofacial Pain Unit. Teknon Medical Center. Barcelona (Spain). Coordinator/Researcher of Institut d´Investigació Biomédica de Bellvitge (IDIBELL), Barcelona, Spain

## Abstract

**Background:**

The aim of this study was to evaluate the clinical and microbiological impact of an antibacterial suture (Monocryl® Plus) in the surgical removal of I3M.

**Material and Methods:**

A “split-mouth”, prospective pilot clinical study was designed involving 20 patients programmed for the surgical removal of I3M. Each side was randomly sutured with Monocryl® Plus or silk suture and removed for microbiological study 72 hours and 7 days after surgery. Presence of SSI, wound bleeding and the degree of discomfort associated with each type of suture material (scored by means of a visual analog scale) were evaluated. The level of contamination of each material was observed under the scanning electron microscope.

**Results:**

Wound bleeding upon suture removing was slightly greater after 72 hours and 7 days with black silk suture, though the differences were not statistically significant (*p*=0.752 and *p*=0.113, respectively). Patient discomfort was very similar with both types of suture material (*p*=0.861). Only one case of SSI was recorded with black silk suture after 72 hours. Microbiologically, the antibacterial suture showed a lesser presence of microorganisms (*p*<0.001, at 72h and *p*=0.033 at 7th day, respectively). The most common bacterial species included grampositive cocci (*Streptococcus viridans* group, *Neisseria spp*., Coagulasenegative *Staphylococcus* and *Peptostreptococcus*), gramnegative cocci (*Veillonella*), grampositive Bacilli (*Lactobacillus*), and gramnegative Bacilli (*Prevotella*).

**Conclusions:**

The greatest antibacterial effect of Monocryl Plus suture was observed after 72 hours. According to most authors, there is no doubt that this antibacterial suture can provide little safety in the control of SSI.

**Key words:**Antibacterial suture, monocryl® plus, vicryl® plus, third molar surgery, postoperative infection, surgical site infection (SSI).

## Introduction

The surgical extraction of impacted third molars (I3M) still remains one of the most commonly performed surgical procedures in Oral Surgery. Surgical site infection (SSI) is among the postoperative local complications that may arise in this surgical procedure. Postoperative infection rate after I3M extraction is around 5% (1). The incidence of SSI is related to intrinsic patient factors (immune-depression, diabetes mellitus, local or systemic infections, etc.) and extrinsic factors (such as smoking, surgical antiseptic measures, wound contamination in clean, contaminated or dirty surgeries, etc) (2). The implantation of sutures or other devices (such as joint prostheses, coronary stents) is also a risk factor for SSI (3). It has been postulated that the number of bacteria required for the development of SSI is about 100,000 times lower in the presence of suture material (4). For over two decades attempts have been made to develop sutures with anti infectious properties. Pharmacologically active substances have also been incorporated on the surfaces of urethral catheters (5), coronary stents (6) or intraocular lenses (7). Antibacterial sutures composed of polyglactin 910 (Vicryl® Plus Antibacterial suture), polyglecaprone 25 (Monocryl® Plus Antibacterial suture) and polydioxanone (PDS® Plus Antibacterial suture) with coated triclosan have been also developed (8-10). Different experimental studies have shown an important reduction in the number of microorganisms (including gram positive and gram negative species) in the region of the surface of these sutures (11-13). Clinical studies in different surgical specialties have demonstrated a relative decrease in SSI (14), and have shown better results in terms of complications commonly seen in the postoperative period (15). Despite the low incidence of SSI after the surgical extraction of I3M, the oral cavity is a highly contaminated area. The aim of this study was to compare the antibacterial effect of Monocryl® Plus suture with silk suture, analyzing the microbiological differences in terms of colonies forming units organisms and species.

## Material and Methods

A “split-mouth” prospective clinical controlled study was designed involving patients treated at the Oral Surgery and Implantology Department of the Faculty of Dentistry at the University of Barcelona (UB), Spain, for the surgical removal of I3M. The study was approved by the Clinical Research Ethics Committee of the UB. Required sample size was estimated at 16 subjects to detect a difference of half the colony forming units in the test group using G*Power software and a paired t-test (effect size dz= 1; α=0.05; 1-β=0.95). Sample size was augmented to 20 subjects to compensate for possible losses. Surgery was performed on twenty healthy patients (ASA I or II) of both sexes aged between 16-45 years. Patients with systemic disease (immune-depression, active infection, diabetes mellitus, hemostatic alterations) were excluded, as were pregnant women, drug abussers, patients with moderate alcohol consumption. Written informed consent was obtained from each patient, as a requirement for participation in the study. Four third year resident surgeons carried out the operations, while other 2 carried out the postoperative clinical follow-up visits. All patients underwent the removal of their 4 third molars presenting similar impaction under conscious sedation provided intravenously by bolus dose of 1.53 mg of midazolam, perfusion pump of 34 mg/kg/hour of propofol and 56 μg/kg/hour of remifentanil. The local anesthetic used was 4% articaine with epinephrine 1:100,000. At least four simple stitches of poliglecaprone 25 suture with triclosan 3/0 suture (Monocryl Plus Antibacterial suture, Ethicon, Somerville, New Jersey, USA), were placed on one side and other four of braided natural black silk 3/0 suture (Suturas Aragó, Laboratorios Aragó S.A., Barcelona, Spain) on the other side. Allocation of each type of suture was performed using a permutation table. The different color of the filaments precluded operator and patient blinding with respect to the type of material used on each side. All patients received instructions on oral hygiene consisting of tooth brushing and cleaning of the surgical wounds with physiological saline rinses three times a day. Postoperative medication consisted of amoxicillin 750 mg every 8 hours for 7 days; sodium diclofenac 50 mg every 8 hours and metamizol magnesium 575 mg every 6 hours during 4 days as rescue analgesia. The patients rated the level of discomfort with suture using a visual analog scale (VAS) scored from 0 to 100 mm during the 7 days after surgery. The clinical variables subjectively registered by the two operators were the presence of bleeding and surgical wound suppuration upon removing the sutures 72 hours and 7 days after surgery.

- Sample processing

One stitch from each operated side was removed 3 and 7 days post operatively, in each patient. Each suture sample was collected in a AMIES semisolid medium and analised in a microbiology laboratory. One linear centimeter of each stitch was sectioned, inoculated in 1 ml of sterile physiological saline and vortexed for 5 minutes to release the microorganisms adhered to the suture material. One microliter of the suspension was seeded with a different culture media and under different atmosphere and temperature conditions. Columbia Agar plates with 5% sheep blood under 5% CO2 at 35°C were used for aerobic bacteria during four days. Yeasts were isolated using Sabouraud Agar plates with gentamycin and chloramphenicol at 35°C during four days. Anaerobic bacteria in turn were isolated using Schaedler Agar plates at 35°C during 7 days. Following the incubation process, the colonies on each plate were counted per colony forming units (cfu/cm/ml). Identification was based on the morphological characteristics, nutritional requirements and biochemical tests. In relation to the latter, the following systems were used: API 20E for *Enterobacteria*, API 20NE for nonfermenting gramnegative bacilli, API NH for *Neisseria spp. and Haemophilus spp*., API Coryne for *Corynebacteria*, API 20 A for anaerobes, Api Rapid ID32 Strep for *streptococci* and Api C AUX for yeasts.

- Scanning electron microscopy (SEM)

Examination of suture materials was carried out under a FEI Quanta™ 200 Scanning Electron Microscope (FEI, Hillsboro, USA) in high vacuum mode and 20 kV, with a spot size of 4 to ensure improved resolution of the magnified images.

- Statistical analysis

Calculation of the differences in the total counts of the microorganisms isolated from both types of suture material was carried out using the nonparametric Wilcoxon signed rank test. The nonparametric Kendall’s W test was used to detect differences between the different isolated species individually. Calculation of the differences in the VAS pain scores was based on analysis of variance (ANOVA) for repeated measures, while the presence of bleeding was assessed with the chisquare test. The Statistical Package for the Social Sciences version 17.0 (SPSS v.17.0; SPSS, Chicago, USA) was used for the statistical analysis, accepting statistical significance for *p*<0.05.

## Results

**Figure 1:**
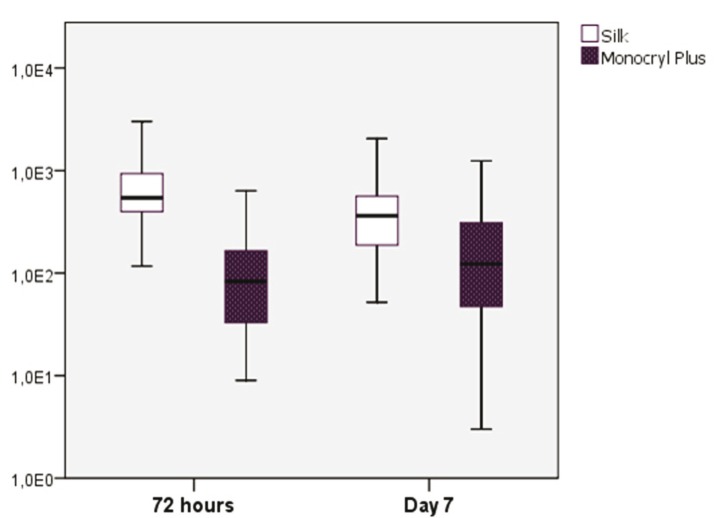
Mean bacterial counts with both sutures, after 3 and 7 days. The ordinates axis exponentially show the counts in cfu/cm/ml.

**Figure 2:**
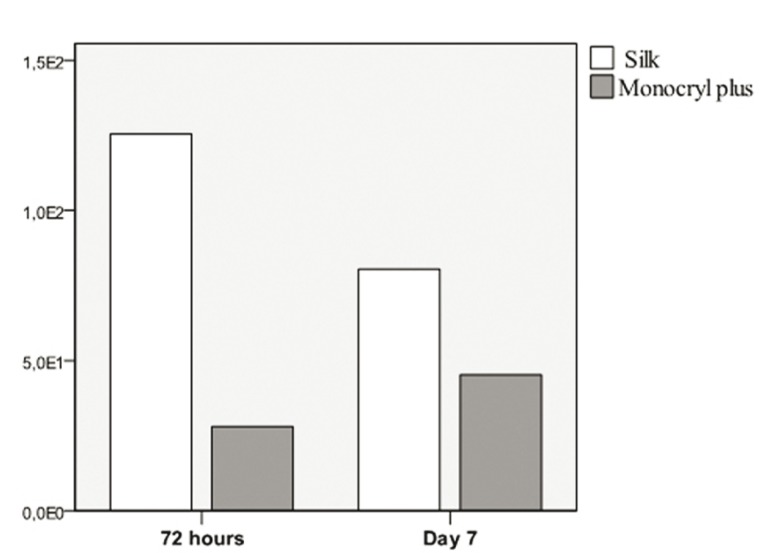
Mean pathogen counts for both sutures, after 3 and 7 days. The ordinates axis exponentially shows the mean counts in cfu/cm/ml.

**Figure 3:**
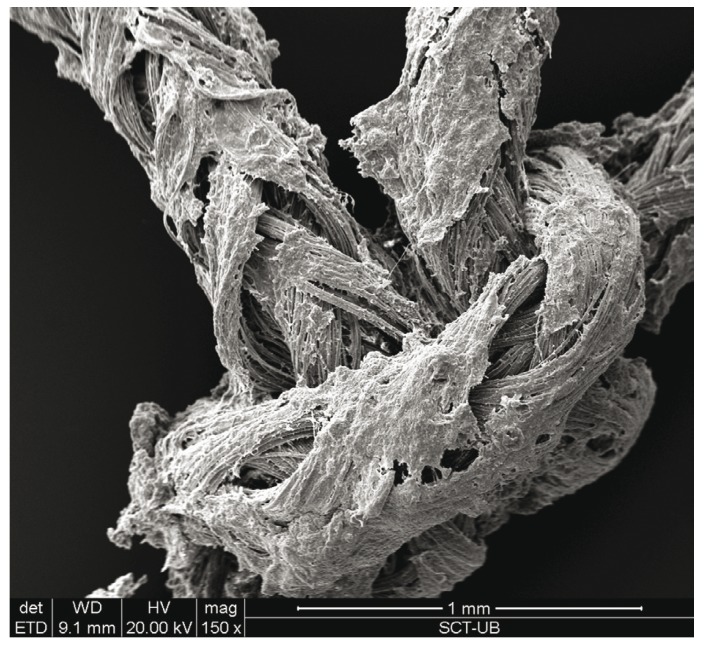
The natural silk suture knot material seen under the scanning electron microscope, 150x.

**Figure 4:**
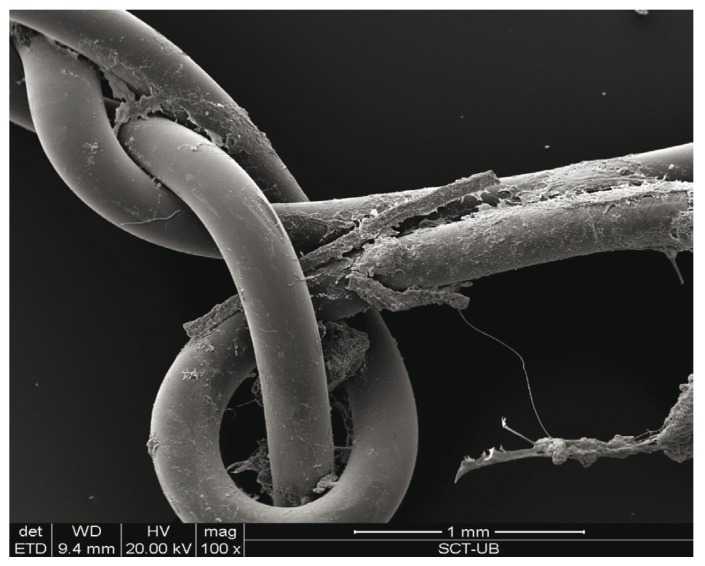
The Monocryl plus suture knot material seen under the scanning electron microscope, 100x.

The study sample consisted of 10 men and 10 women, aged between 18 and 35 years, with a mean age of 23.6 years (standard deviation (SD) of 4.77). Wound bleeding upon suture removal was slightly more frequent with silk suture though the differences were not statistically significant (*p*=0.752 and *p*=0.113, respectively). The degree of discomfort (scored by the patients on the VAS) was very similar on both sutures (*p*=0.861), though with silk suture was slightly greater during the entire postoperative period. On day 3 there was one case of surgical wound infection with silk suture. The mean microorganisms count after 3 days was considerably lower with the antibacterial suture ( Table 1). In contrast, by day 7 the difference between the two sutures had decreased ( Table 2), though the differences between the two types of suture were statistically significant (*p*<0.001 and *p*=0.033) (Fig. 1). According to these results, there was mean bacterial reduction of 83% and 65%, respectively. In relation to the total microorganisms count, aerobes were considerably more prevalent than anaerobes (1292 cfu/cm/ml; SD of 1230 and 349 cfu/cm/ml; SD of 368, respectively) (*p*=0.001). Silk suture showed significantly higher values for both aerobes and anaerobes. Among the most frequently isolated species, mention must be made of (*Streptococcus viridans* group (*S. mitis, S. oralis, S. salivarius, S. parasanguis, S. sanguinis, S. anginosus and S. intermedius*) Coagulase-Negative *Staphylococcus, Peptostreptococcus spp., Veillonella spp., Lactobacillus spp. and Prevotella spp*. In only one case was the presence of Candida spp. detected, with lower counts in the case of the antibacterial suture. In general, Monocryl® Plus yielded a lower count for almost all the isolated species. However, the differences were only statistically significant after 3 days (125 cfu/cm/ml; SD of 179 for silk suture and 28 cfu/cm/ml; SD of 42 for Monocryl® plus suture) (*p*=0.013). After 7 days the differences had decreased (80 cfu/cm/ml; SD of 169 and 45 cfu/cm/ml; SD of 116, respectively), though not significantly (*p*=0.197). Among the pathogenic organisms isolated, mention should be made of gramnegative bacilli (*Citrobacter freundii complex, Prevotella spp., Prevotella disiens and Fusobacterium spp*.), anaerobic grampositive cocci (*Peptostreptococcus spp*.) and other unidentified strict anaerobes. The difference between these organisms and the commensal flora was significantly favorable to the nonpathogenic species in both black silk (*p*=0.012) and in Monocryl® Plus suture (*p*=0.003). The most important reduction in microorganisms in the antibacterial suture corresponded to the commensal bacteria (*p*=0.001). However, the presence of pathogenic microorganisms was also less evident in this suture after 72 hours and 7 days - though not statistically significant (Fig. 2). The scanning electron microscopic study showed the differences in bacterial colonization between the two types of suture material. Figures 3,4 shows the largest presence of microorganisms and cellular detritus to be contained in a biofilm of extracellular polysaccharides at the silk suture knot respect to study suture.

**Table 1:**
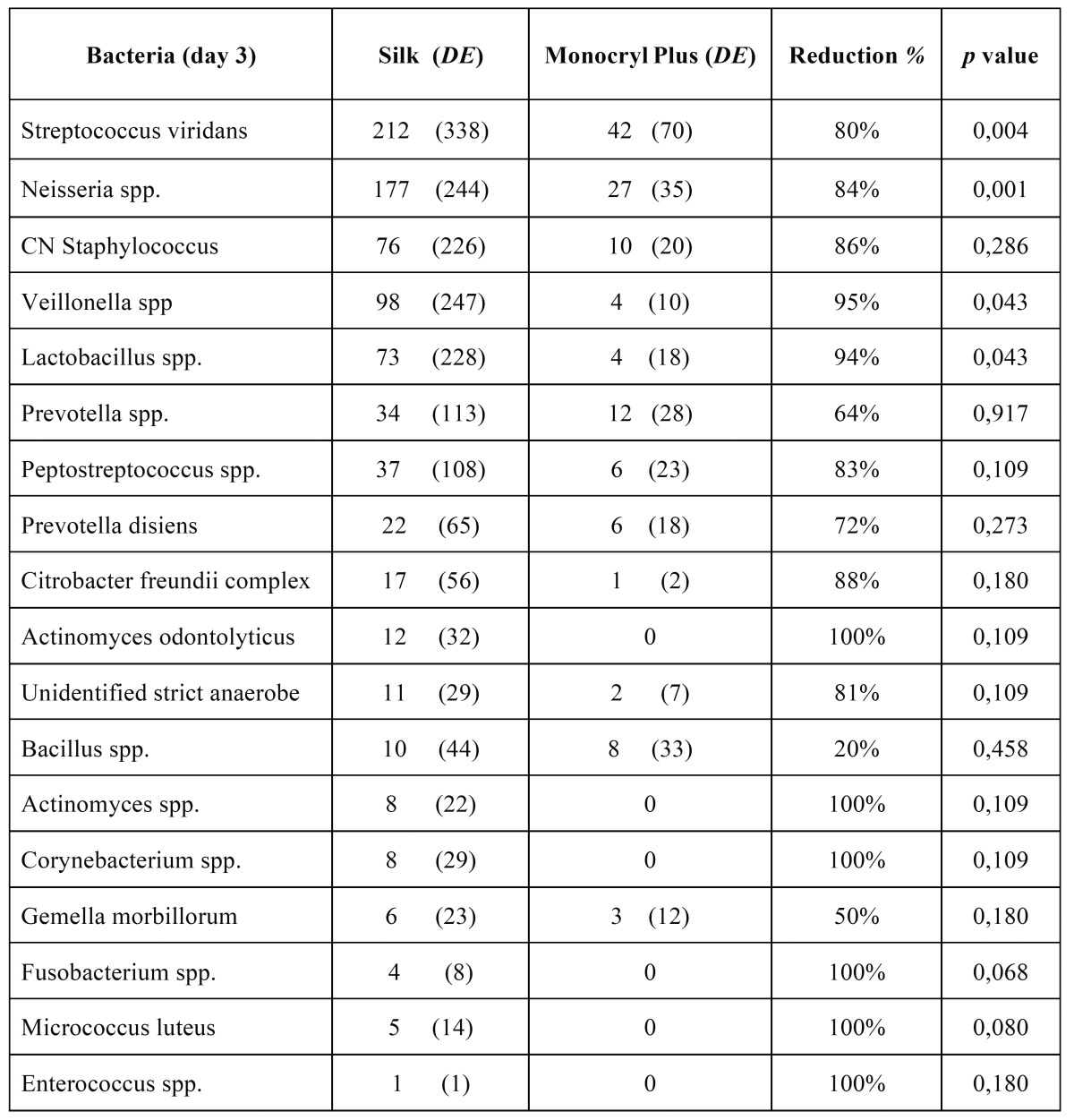
Mean counts of the isolated bacterial species with both suture materials 3 days after surgery. *CN Staphylococcus* = *Coagulase-negative Staphylococcus*.

**Table 2:**
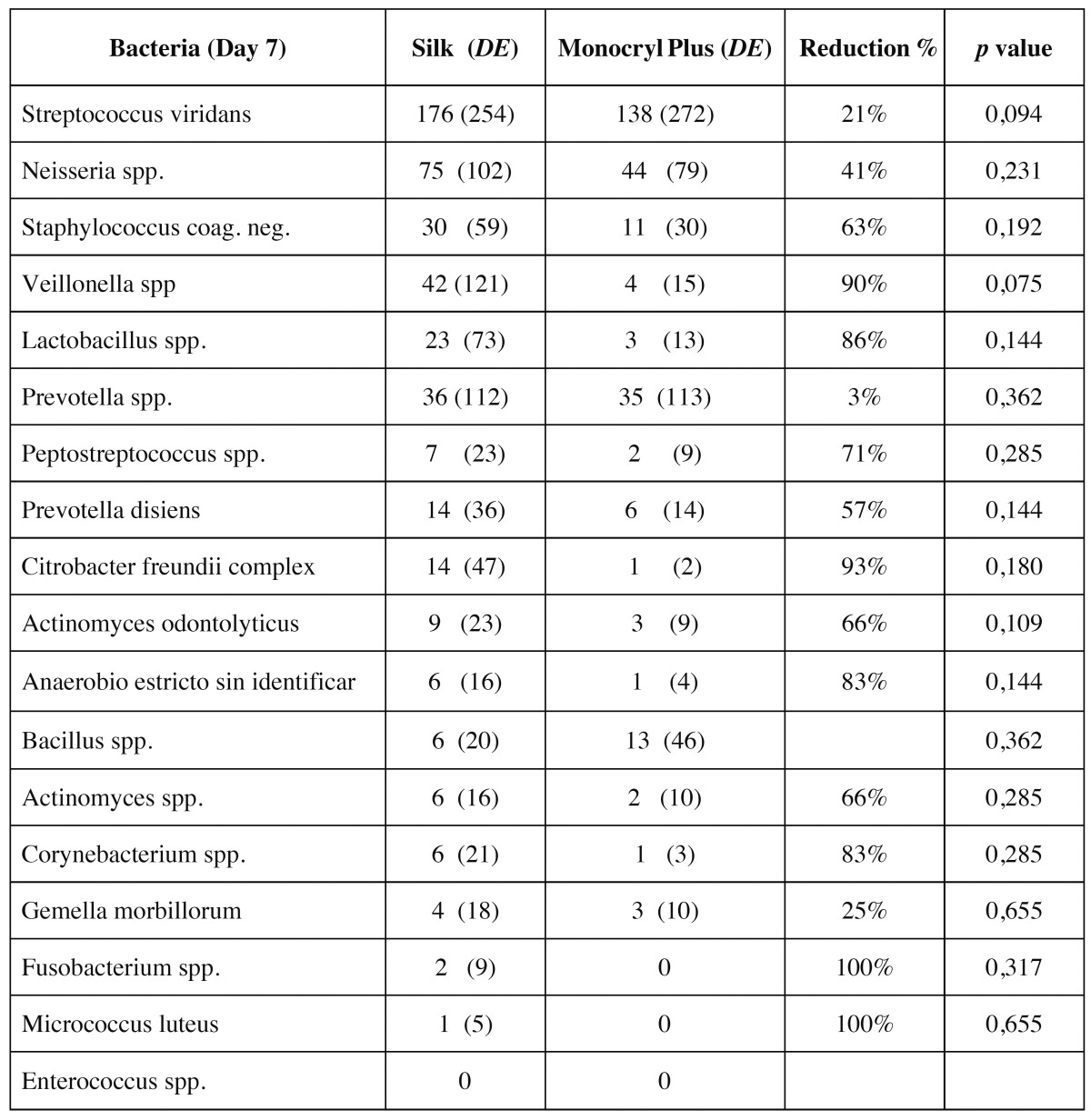
Mean counts of the isolated bacterial species with both suture materials 7 days after surgery. *CN Staphylococcus* = *Coagulase-negative Staphylococcus*.

## Discussion

Surgical site infection (SSI) is the third most common cause of nosocomial infections, and the most among surgical patients (16). Two-thirds of all cases of SSI appear in the zone of the incision. This probability is even greater in the presence of suture material (17). It has been estimated that with conventional sutures (such as the natural black silk), barely 100 cfu would be needed to induce SSI (4). Many methods have been studied to decrease the incidence of surgical site infection, although some are uncontrollable others can be controlled. One of these methods is the use of sutures coated with triclosan. In 2002, the United States Food and Drug Administration (FDA) authorized the use of polyglactin 910 coated with triclosan (Vicryl® Plus, Antibacterial suture). Most studies conducted with sutures of this kind report a decrease in the amount of microorganisms sticked to their surface. However, Venema *et al*. (18), in an *in vitro* study with Vicryl® Plus suture, recorded no bacterial inhibition zone around the suture with either *Streptococcus sanguis* PK1889 or microorganisms from a human saliva sample. In contrast, animal studies have obtained favorable results. Storch *et al*. (13) reported a reduction of 96.7% with Vicryl® Plus suture after 48 hours in strains of *S.aureus*. Ming *et al*. (8), in a similar study but using Monocryl® Plus suture, recorded a bacterial reduction in the order of 3.4 log and 2 log in strains of *S.aureus* and *E. coli*, respectively. Gómez Alonso *et al*. (11) in turn obtained a reduction of about 87% with Vicryl® Plus suture previously infected with *S. epidermidis* and *E. coli*. Lastly, Marco *et al*. (19), in a study using rats, reported a 66% reduction in cultures positive for *S.epidermidis*. This is the first human study to date of the antibacterial action of Monocryl® Plus mono filament suture based on a quantitative and qualitative analysis of the microorganisms. This suture provide the support necessary to maintain wound-edge approximation during the critical healing period (5-7 days after surgery) due to the high initial breaking strength, pass smoothly through fascia to minimize tissue trauma as consequence of its mono filament design and polymer properties that minimize drag force and elicit only a slight tissue reaction during absorption. Furthermore, protect against colonization of the suture by organisms commonly associated with SSIs. In our study colonization rate was 83% and 65% lower than with silk suture after 3 days (the mean bacterial count being up to 5fold lower than with silk suture) and 7 days (2fold lower) respectively. However, differences in scanning electron microscope images obtained with both sutures, were even clearer. The complex adhesion mechanisms of the microorganisms that inhabit the oral cavity, the structural characteristics of silk suture braiding, and the absence of sonication during sample processing probably would explain this observation (18,20). Triclosan is an antiseptic component with bacteriostatic action. At low concentrations, inhibits the growth of many nonsporulating gram positive and gram negative bacterial species. The amount added to these sutures reaches 1.5 μg/cm, and the range of minimum inhibitor concentrations (MICs) against the microorganisms that inhabit the oral cavity is 0.0017.8 μg/ml (18). In our study, the presence of triclosan in Monocryl® Plus was associated to a significant reduction of most microorganisms isolated during the first 2-3 days, though by day 7 after surgery this reduction was less notorious. In an in vitro study conducted by Ming *et al*. (21), the diffusion pattern was maintained for up to 21 days. The opposite effect was recorded with silk suture, however - a larger number of bacterial colonies being observed after 3 days (778 cfu/cm/ml) than after 7 days (468 cfu/cm/ml) of suture placement. This phenomenon appears to be related to the inability to maintain adequate oral hygiene, as a result of the limitation in mouth opening, pain and swelling in the surgical zone still present on the third postoperative day. The bacterial flora responsible for odontogenic infections is diverse. In the present study, the number of bacterial species and colonies was greater in the case of aerobic microorganisms (n = 12) than for anaerobes (n = 5), presumably as a result of selection of the culture media. On the other hand, this circumstance may possibly explain the relatively low incidence of infectious complications recorded. Studies on oral and cervicofacial infections of odontogenic origin have also detected a predominantly aerobic flora (22,23). In the present study, the use of Monocryl® Plus suture yielded the lowest counts of both aerobic (*p*=0.001) and anaerobic organisms (*p*=0.017). *Streptococci* belonging to the viridans group were most prevalent species with both sutures, followed by *Neisseria spp*. and coagulasenegative *Staphylococcus*. The unexpected presence of CoagulaseNegative *Staphylococcus* (8.3% with silk sutures and 5.3% in the case of Monocryl® plus) may have been due to skin contamination of the suture filaments. The most important difference after 72 hours corresponded to the *Streptococcus viridans* group (*p*=0.001). The non pathogenic microorganism counts were greater than the pathogens for both sutures, though the antibacterial suture had the lowest pathogen counts. With Monocryl® Plus there was a mean reduction of 90% for *Citrobacter freundii*, 78% for *Peptostreptococcus*, 33% for *Prevotella spp*., 64% for *Prevotella disiens*, 82% for the unidentified anaerobes, and 100% for *Fusobacterium spp*. The main objective of antibacterial sutures has been to reduce the SSI rate by inhibiting bacterial growth onto the surface of the suture material. To date, the results of the studies made with this suture material in humans have not been as good as expected. Studies in different medical and surgical areas (neurosurgery (14), chest surgery (24), general surgery (25), general pediatric surgery (15), plastic and reconstructive surgery (26,27) and oral surgery have evaluated antibacterial sutures. We recorded a total of 8 studies, of which three (14,24) reported a reduction in the number of cases of SSI. Other authors however, have reported no advantage with the use of these antibacterial sutures (25,27). Ford *et al*. (15) and Deliaert *et al*. (26) recorded no SSI with the sutures studied in their series, and in the present study there was a single case of wound infection with natural silk. In the studies published by Mingmalairak *et al*. (25) and Chen *et al*. (27), surgery was carried out in highly contaminated areas. In contrast, the operations carried out in our series were regarded as clean or clean-contaminated surgery. Probably, in our study antibiotic prescription in the postoperative period may have masked the effect associated to antibacterial suture use. Inflammatory response measured by the presence of wound bleeding was similar with both types of suture. Surgical site inflammation after the extraction of I3M is difficult to avoid in the first 2-3 days In contrast, after 7 days, wound healing is more advanced. Differences in bleeding in our study were not significant, though either the effects of the remaining traces of triclosan or the lesser bacterial aggregation associated with the use of Monocryl® plus caused the inflammatory reaction to be less pronounced with the antibacterial suture material after 7 days. Some experimental studies have reported a reduction in inflammatory response and lesser bacterial colonization with standard Monocryl® suture (28,29). No statistically significant differences were recorded in the level of pain experienced by the patients with the two suture materials. It should be noted that with the Monocryl® Plus suture the patients reported greater discomfort due to the irritation caused by the stiffness of the suture extremities. In contrast, Ford *et al*. (15) found polyglactin 910 with triclosan to result in less pain, though the areas in which this suture material was used were less sensitive than the oral cavity. These results may have been conditioned by the absence of blinding to the suture material employed, since the filaments were of different colors.

## Conclusions

The most significant antibacterial effect of Monocryl® Plus suture occurred in the first 3 days. Nevertheless, 7 days after surgery there was some bacterial reduction vs silk suture. Commensal species (*Streptococcus viridans* group) were more frequently isolated than pathogenic organisms (*Prevotella spp., Fusobacterium spp*.). The postoperative infection rate was close to zero per cent with both sutures. For this reason it would be advisable to carry out a clinical study with a larger sample of patients in order to determine whether antibacterial sutures effectively contribute to lessen surgical site infections in patients subjected to lower third molar extractions. In extraction of impacted third molars, Monocryl® Plus suture does not seem to improve substantially of the rate of SSI.
